# Passive administration of purified secretory IgA from human colostrum induces protection against *Mycobacterium tuberculosis* in a murine model of progressive pulmonary infection

**DOI:** 10.1186/1471-2172-14-S1-S3

**Published:** 2013-02-25

**Authors:** Nadine Alvarez, Oscar Otero, Frank Camacho, Reinier Borrero, Yanely Tirado, Alina Puig, Alicia Aguilar, Cesar Rivas, Axel Cervantes, Gustavo Falero-Díaz, Armando Cádiz, María E Sarmiento, Mohd Nor Norazmi, Rogelio Hernández-Pando, Armando Acosta

**Affiliations:** 1 Department of Molecular Biology. Finlay Institute. Center of Research – Producction of Vaccines. Ave. 27 No. 19805, La Lisa. Ciudad de la Habana, Cuba. AP. 16017, CP 11600; 2 Experimental Pathology Section, Department of Pathology, National Institute of Medical Sciences and Nutrition “Salvador Zubiràn”, D.F. Mexico. CP 14 000; 3 Enterprise of Production of Serum and Hemoderivates “Adalberto Pesant González”. Ave 51 No.33 235 km 19 medio ½. Arroyo Arenas, La Lisa. Ciudad de la Habana, Cuba. CP 13400; 4 School of Health Sciences, Universiti Sains Malaysia, 16150 Kubang Kerian, Malaysia; 5 Institute for Research in Molecular Medicine, Universiti Sains Malaysia, 16150 Kubang Kerian, Malaysia

## Abstract

**Background:**

Immunoglobulin A is the most abundant isotype in secretions from mucosal surfaces of the gastrointestinal, respiratory and genitourinary tracts and in external secretions such as colostrum, breast milk, tears and saliva. The high concentration of human secretory IgA (hsIgA) in human colostrum strongly suggests that it should play an important role in the passive immune protection against gastrointestinal and respiratory infections.

**Materials and methods:**

Human secretory IgA was purified from colostrum. The reactivity of hsIgA against mycobacterial antigens and its protective capacity against mycobacterial infection was evaluated.

**Results:**

The passive administration of hsIgA reduces the pneumonic area before challenge with *M.* tuberculosis. The intratracheal administration of *M. tuberculosis* preincubated with hsIgA to mice greatly reduced the bacterial load in the lungs and diminished lung tissue injury.

**Conclusions:**

HsIgA purified from colostrum protects against *M. tuberculosis* infection in an experimental mouse model.

## Introduction

Mucosal infections caused by intracellular pathogens induce cellular immune responses [1], mediated by CD4^+^ and CD8^+^ T cells, normally accompanied by the production of antibodies including the synthesis of human secretory immunoglobulin A (hsIgA), which provides a first important line of defense against invasion of pathogens into tissues [2]. IgA antibodies are not only present in external secretions, but also exert antimicrobial activities in epithelial cells during their passage through the epithelium. These antibodies represent the predominant class of immunoglobulin in external secretions and provide a specific immunological protection in all mucosal surfaces, blocking the entry of pathogenic agents [3]. Mycobacterial infection takes place primarily through the respiratory system. However, the role of IgA in the immune response against mycobacteria has not been well described.

## Materials and methods

Human secretory IgA was purified from healthy women colostrum with the hospital's consent, by a combination of chromatographic methods using anion exchange chromatography in DEAE-Sepharose Fast Flow matrix, and molecular exclusion chromatography using Superose 6 prep grade matrix, according to the method described by Goil *et al*., 1998 [4].

The purified hsIgA was analysed by SDS-PAGE (acrylamide gel 12.5 %) in reduced conditions [5]. The reactivity of hsIgA was determined against mycobacterial antigens using cell preparations of *M. bovis* BCG and a whole cell lysate of *M. tuberculosis* by Western Blotting according to Towbin [6].

The protective capacity of hsIgA was evaluated against *M. tuberculosis* infection in BALB/C mice, distributed in 3 groups of 20 mice each one. The non-treated (NT) group: animals were infected with 2.5 x 10^5^ CFU of *M. tuberculosis* in 100 μL of saline solution by intratracheal route. The hsIgA group: animals were inoculated by the intranasal route with the hsIgA (1mg in 50 μL of saline solution, 25 μL in each nostril) and challenged two hours later with 2.5 x 10^5^ CFU of *M. tuberculosis* by the intratracheal route. The preincubated hsIgA (Preinc) group: animals were challenged intratracheally with 2.5 x 10^5^ CFU *M. tuberculosis* previously incubated with 1 mg of the hsIgA during 4 hr at room temperature.

Five mice from each group were sacrificed at 1, 7, 30 and 60 days after challenge with *M. tuberculosis*. To assess the efficiency of bacilli clearance by the immunoglobulin treatment, bacilli load was determined by assessing the CFU of *M. tuberculosis* in lung homogenates after sacrifice. CFU were counted by plating 10-fold serial dilutions of the homogenates onto Middlebrook 7H10 nutrient agar (Difco, USA) plates and incubated at 37°C. Colonies were counted twice under a stereoscopic microscope after 14 days of incubation. In addition, lung tissues sections were stained with hematoxylin and eosin [7]. The pneumonic areas were measured and analyzed using Leica Q-win system software (Leica Microsystems Imaging Solutions LTD, Cambridge, UK, 25x). The results of the CFU in lungs in all groups and time intervals were studied using ANOVA and a post hoc Tukey multiple comparison procedure. *p*-values under 0.05 were considered statistically significant. All data was analysed using GraphPad Prisma 4 Software.

## Results and discussion

The fraction corresponding to specific hsIgA was eluted with 100 mM sodium phosphate buffer at pH 6.4, showing a bimodal peak, taking into account that its isoelectric point is 6.5 [8]. Then, applying the sample on a column packed with Superose 6 prep grade, equilibrated with PBS, the IgA was consistently eluted in a unique symmetrical peak. In order to asses the purity of the sample, the purified hsIgA was analyzed by SDS-PAGE on a 12.5 % acrylamide gel under reduced conditions, showing only the 3 characteristic bands corresponding to the molecule of hsIgA: secretor component (SC), heavy chain (HC) and light chain (LC).

**Figure 1:**
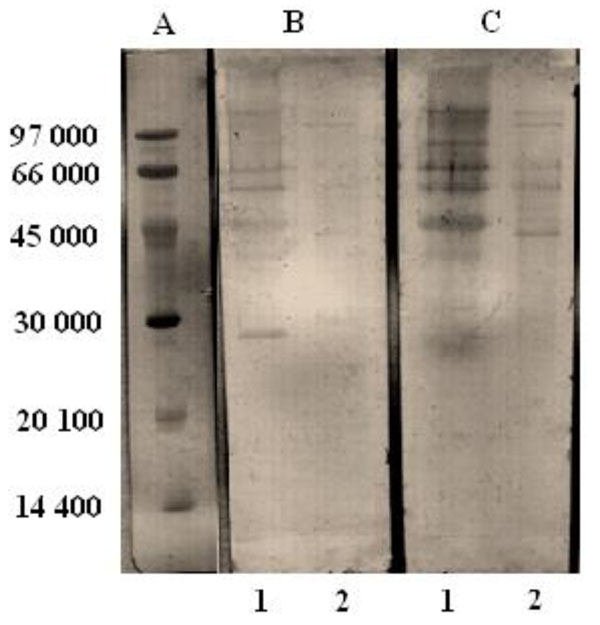
Western blot of whole cell preparation of *M. bovis* BCG (1) and whole cell lysate of *M. tuberculosis* (2) samples, separated by SDS-PAGE 12.5% acrylamide gel. A: Molecular weight markers (Pharmacia), B: human colostrum (1/20); C: purified human secretory IgA (100µg/ml).

With the aim of studying the reactivity of the hsIgA against specific antigens of mycobacteria, the samples were analyzed by Western Blotting, using Intacglobin, an immunoglobulin formulation previously shown to have high reactivity against *M. bovis* BCG and *M. tuberculosis* antigens, as a positive control [9]. The strip incubated with purified hsIgA, showed higher recognition than the one incubated with human colostrum, and was almost comparable to that of Intacglobin. It is noteworthy that the reactivity of both products was higher against the antigens of *M. bovis* BCG than *M. tuberculosis* antigens (Fig.1). The recognition of mycobacterial antigens by the hsIgA preparation obtained from human colostrum, could be due to previous, latent or current tuberculosis infection or prior vaccination with BCG and or exposure to environmental mycobacteria.

**Figure 2:**
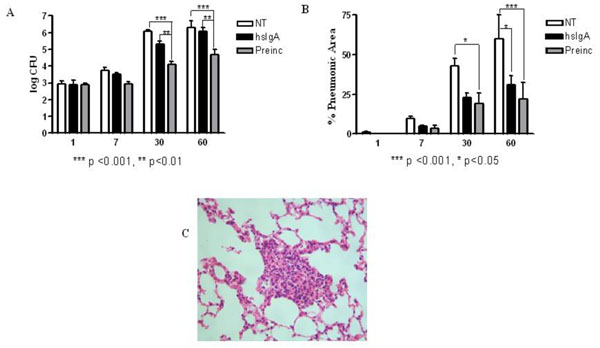
Determination of bacterial load (A) and pneumonic area (B) in lungs of mice which were untreated (NT) and those treated with hsIgA (hsIgA), after challenge with *M. tuberculosis* H37Rv by intratracheal route 2 hrs after inoculation. Another group received *M. tuberculosis* preincubated with hsIgA (preinc). Granulomas of preincubated group 2 months after challenge with *M. tuberculosis*, visualized by hematoxilin-eosin staining (25x) (C). The morphometric study was carried out with light microscopy using Leica Q-win System Software. All data was analysed using GraphPad Prisma 4 Software. Each bar represents the mean of three samples ± SD.

The reactivity demonstrated by hsIgA against mycobacterial antigens was an important starting point for performing the challenge tests in order to assess its protective capacity against mycobacterial infection, because the prophylactic effect of the administration or pre-incubation of *M. tuberculosis* with hsIgA has not been previously explored. The present study described the potential prophylactic effect of intranasal administration of hsIgA before challenge with *M. tuberculosis* via the intratracheal route. Administration of hsIgA 2 hr before challenge resulted in a significant decrease in the CFU in lungs compared to the control group at all times points (p<0.05) (Fig.2). Inoculation of mice with *M. tuberculosis* preincubated with hsIgA resulted in significant decrease in the CFU of lungs, compared with the non-treated group, two months post-challenge (p<0.05) (Fig.2). Furthermore, the lungs of infected mice treated with purified hsIgA or those inoculated with preincubated *M. tuberculosis*, showed better organized granulomas and reduced pneumonic areas compared to the control group (Fig. 2C). These findings are in accordance to the reduced bacterial load as well as lower morphometric and histopatological changes observed in the lungs of mice treated with an IgA monoclonal antibody against 16 kDa protein of *M. tuberculosis* (TBA61) [10].

HsIgA is structurally and functionally suitable for the mucosal environment. It neutralizes antigens and viruses, promotes microorganism agglutination, facilitates antigenic exclusion and prevents the adherence of pathogens to epithelial mucosal surfaces [11]. HsIgA antibodies can enhance the adherence of bacteria and other antigens to mucus because of the mucophilic properties of their bound SC [12]; thereby promoting clearance of immune complexes by respiratory ciliary movement and intestinal peristalsis. IgA antibodies recognizing mycobacterial surface constituents could thus have an additional targeting opportunity to influence the course of intracellular infection, by virtue of interfering with the interactions between opsonised bacteria and phagosomal membrane. Such interactions could negatively influence bacterial survival, since close apposition with the phagosomal membrane is thought to be important for the capacity of *M. tuberculosis* bacilli to inhibit phagosomal maturation and fusion with lysosome [13]. The hsIgA action could involve a number of mechanisms: e.g. (a) superior homing to the lungs following intranasal (but not intravenous) delivery [14]; (b) antibody-dependent cellular cytotoxicity [15]; and (c) stimulation of antigen presenting cells, required for T-cell activation [16]. The results presented here support the increased interest in the role of antibodies in controlling intracellular microbial infections and particularly for exploiting the IgA isotype for protection.

Our results demonstrated for the first time the prophylactic effect of mucosal administration of sIgA obtained from human colostrum in a mouse model of infection with *M. tuberculosis*. In addition, we have demonstrated that, incubation of *M. tuberculosis* with hsIgA could inhibit the infective potential of the pathogen.

## Competing interests

The authors declare that they have no competing financial interests.

## Authors' contributions

All authors have read and approved the final manuscript. NA participated in the purification and characterization of IgA, in the challenge experiments and in data analysis and writing of the manuscript. OO, FC, RB, GF, AC participated in the purification and characterization of IgA. DA, CR, AC, HO, RHP, YT, AP and AA participated in the challenge experiments. MES, MNN, RHP, AA designed the study, participated in data analysis and in writing of the manuscript. 

## Declarations

This article has been published as part of *BMC Immunology* Volume 14 Supplement 1, 2013: Proceedings from Delivery Systems and Current strategies to drug design. The full contents of the supplement are available online at http://www.biomedcentral.com/bmcimmunol/supplements/14/S1
